# Fusion rates based on type of bone graft substitute using minimally invasive scoliosis surgery for adolescent idiopathic scoliosis

**DOI:** 10.1186/s12891-023-06134-1

**Published:** 2023-01-14

**Authors:** Jae Hyuk Yang, Hong Jin Kim, Dong-Gune Chang, Seung Woo Suh

**Affiliations:** 1 Department of Orthopedic Surgery, Korea University Anam Hospital, College of Medicine, Korea University, Seoul, Korea; 2 Spine Center and Department of Orthopedic Surgery, Inje University Sanggye Paik Hospital, College of Medicine, Inje University, 1342, Dongil-Ro, Nowon-Gu, Seoul, 01757 Republic of Korea; 3 Department of Orthopedic Surgery, Korea University Guro Hospital, College of Medicine, Korea University, Seoul, Korea

**Keywords:** Adolescent idiopathic scoliosis, Conventional open scoliosis surgery, Minimally invasive scoliosis surgery, Fusion rates, Clinical outcomes

## Abstract

**Background:**

Minimally invasive scoliosis surgery (MISS) is currently introduced on novel technique for surgical treatment of adolescent idiopathic scoliosis (AIS). This study is aimed to evaluate the efficacy of facet fusion in MISS compared to posterior fusion in conventional open scoliosis surgery (COSS) and compare facet fusion rates based on three bone graft substitutes in MISS for adolescent idiopathic scoliosis (AIS).

**Methods:**

Eighty six AIS patients who underwent scoliosis surgery were divided into two groups: the COSS group and the MISS group. COSS was performed through posterior fusion with allograft. MISS was applied via facet fusion with three bone graft substitutes. The MISS group was further divided into three subgroups based on graft substitute: Group A (allograft), Group B (demineralized bone matrix [DBM]), and group C (demineralized cancellous bone chips). Fusion rate was measured using conventional radiographs to visualize loss of correction > 10°, presence of lysis around implants, breaks in fusion mass, and abnormal mobility of the fused segment.

**Results:**

The fusion rates showed no significant difference in COSS and MISS groups (*p* = 0.070). In the MISS group, the fusion rates were 85, 100, and 100% in groups A, B, and C, respectively, with no significant difference (*p* = 0.221). There were no statistical differences between groups A, B, and C in terms of correction rate, fusion rate, and SRS-22 scores (*p* > 0.05).

**Conclusions:**

The facet fusion in MISS showed comparable to posterior fusion in COSS with regard to radiological and clinical outcomes. Furthermore, the type of graft substitute among allograft, DBM, and demineralized cancellous bone chips did not affect facet fusion rate or clinical outcomes in MISS. Therefore, MISS showed comparable fusion rate (with no influences on the type of graft substitute) and clinical outcomes to those of COSS in the surgical treatment of AIS.

## Background

Surgical interventions for adolescent idiopathic scoliosis (AIS) should achieve correction, stability, and meticulous spine arthrodesis [1]. The advent of pedicle screw instrumentation has resulted in better outcomes in terms of overall correction and improved coronal and sagittal balance [2]. Although instrumentation helps with stabilization, the success of fusion is ultimately determined by biological mechanisms that stimulate bones to coalesce to form a solid mass [3–5]. Traditionally, autologous local bone graft tissue obtained from resection of spinous processes and decortication of posterior elements and facet joints has been used as elementary graft material [6, 7]. As the volume of harvested local bone is usually insufficient for long segmental fusion, bone grafts or graft substitutes are frequently added [3, 8]. The outcomes of autologous iliac crest grafts, allografts, and bone graft substitutes have been studied extensively [9, 10]. Autologous iliac crest grafting is considered the gold standard and is the benchmark for comparisons with other graft substitutes [6, 11]. However, due to limited availability and donor site morbidity in 19–30% of cases, surgeons frequently resort to other options [6]. Allografts can be obtained in relatively large quantities and, therefore, represent a viable option. However, the costs, infection rates, potential immunogenicity, and microbial contamination associated with allografts have led to development of bone graft substitutes [11]. Recently, a wide array of bone graft substitutes, such as demineralized bone matrix (DBM), tricalcium phosphates, biphasic phosphate ceramics, and silicated-calcium phosphates, has become available. These possess osteoconductive or osteoinductive properties and can be used as graft expanders, graft enhancers, or graft substitutes. Bone graft substitute use has been associated with fusion rates similar to those of autologous bone grafts [8, 12].

Classically, the main aim of spinal deformity correction surgery is to optimize proper shape and normal range of the spine and to perform stable fixation so that additional problems do not occur in the corrected vertebral body because of the bone graft [13]. However, in recent years, improving cosmesis has become a primary objective of deformity correction. The past decade has brought significant advances in minimally invasive surgery (MIS) for deformity correction; these advances, which have helped spine surgeons achieve their objectives more effectively [14]. However, final clinical outcomes are influenced by the extent of correction and the strength of fusion achieved [15]. The fate of spinal arthrodesis and the optimal choice of bone grafts or graft substitutes with these novel minimally invasive scoliosis surgery (MISS) procedures remain largely undetermined [16]. With MISS, the midline soft-tissue collar is essentially preserved; and the main bed for fusion is provided only by the facets. Hence, study of the fusion rates associated with the novel MISS techniques is necessary before their adoption on a large scale. Our study aimed to investigate the preliminary clinical and radiological outcomes of MISS with facet fusion based on three bone graft substitutes. Furthermore, we compare these outcomes with those of the conventional open scoliosis surgery (COSS) with posterior fusion for AIS using allografts.

## Methods

After obtaining Institutional Review Board approval, we evaluated 86 patients with AIS who underwent posterior correction surgery between 2014 and 2015. This was a retrospective controlled comparative study conducted by a single institution in which spinal deformity corrections were routinely performed. The 86 patients in this study were divided into two groups: the COSS group (patients who underwent COSS) and the MISS group (patients who underwent MISS). Inclusion criteria were 1) patients with AIS, 2) age at the time of surgery: 11 to 18 years, and 3) Cobb’s angle of major curve between 50° and 80°. The exclusion criteria were 1) neuromuscular or congenital scoliosis, 2) anterior procedures for deformity correction, 3) revision surgery, 4) refusal to provide consent to participate in the study, 5) known metabolic disorders or malignancy, 6) mental retardation, and 7) acute local or systemic infection. Patients and parents were informed on the graft materials, and written consent for surgery was obtained. In the COSS group, commercially available freeze-dried corticocancellous allografts were used for posterior fusion. In the MISS group, patients were further divided into three subgroups based on graft materials used for facet fusion: group A used corticocancellous allografts, group B used 100% DBM sterilized by irradiation, and group C used demineralized cancellous bone chips sterilized by irradiation. The subgroup of patients was randomly allocated.

All patients were operated on by a single surgeon (senior author) who had vast experience with COSS. The surgical procedure of COSS was illustrated as shown. The proximal neutral vertebra was the proximal extent of fusion, while the distal extent of fusion was determined using Suk’s guidelines [2]. In the COSS procedure, mono-axial pedicle screws were inserted at each vertebra within the instrumented segment; correction was achieved through a rod derotation (RD) maneuver through a posterior-only approach.

**Fig. 1:**
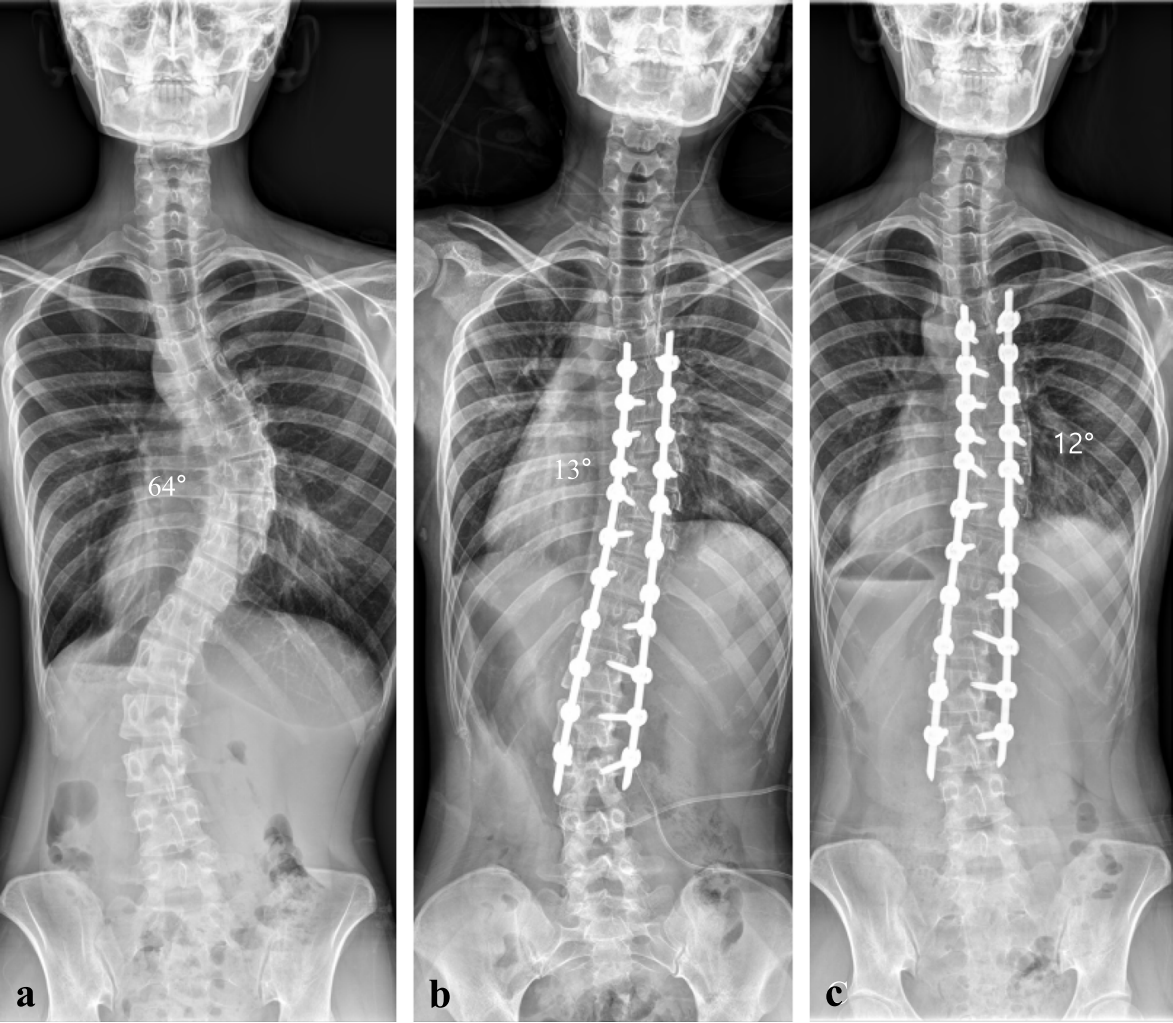
A 15-year-old female patient visited the hospital for progressive spinal deformity. She showed a rip hump and lumbar prominence. The whole-spine anterior–posterior image showed 64° of scoliosis deformity, and the Risser stage was evaluated as Grade 4 (**a**). Considering age, Risser stage, and scoliosis angle, spine correction was performed. The surgery was performed using the minimally invasive scoliosis surgery (MISS) technique, and fusion was performed using the facet fusion technique and allograft bone chips. Postoperatively, the deformity was corrected from 64° to 13° (**b**). On a whole-spine anterior–posterior image taken 2 years after surgery, the corrected scoliosis deformity was maintained at 12°, and reduction loss, segmental fracture, and decompensation were not identified (**c**)

**Fig. 2:**
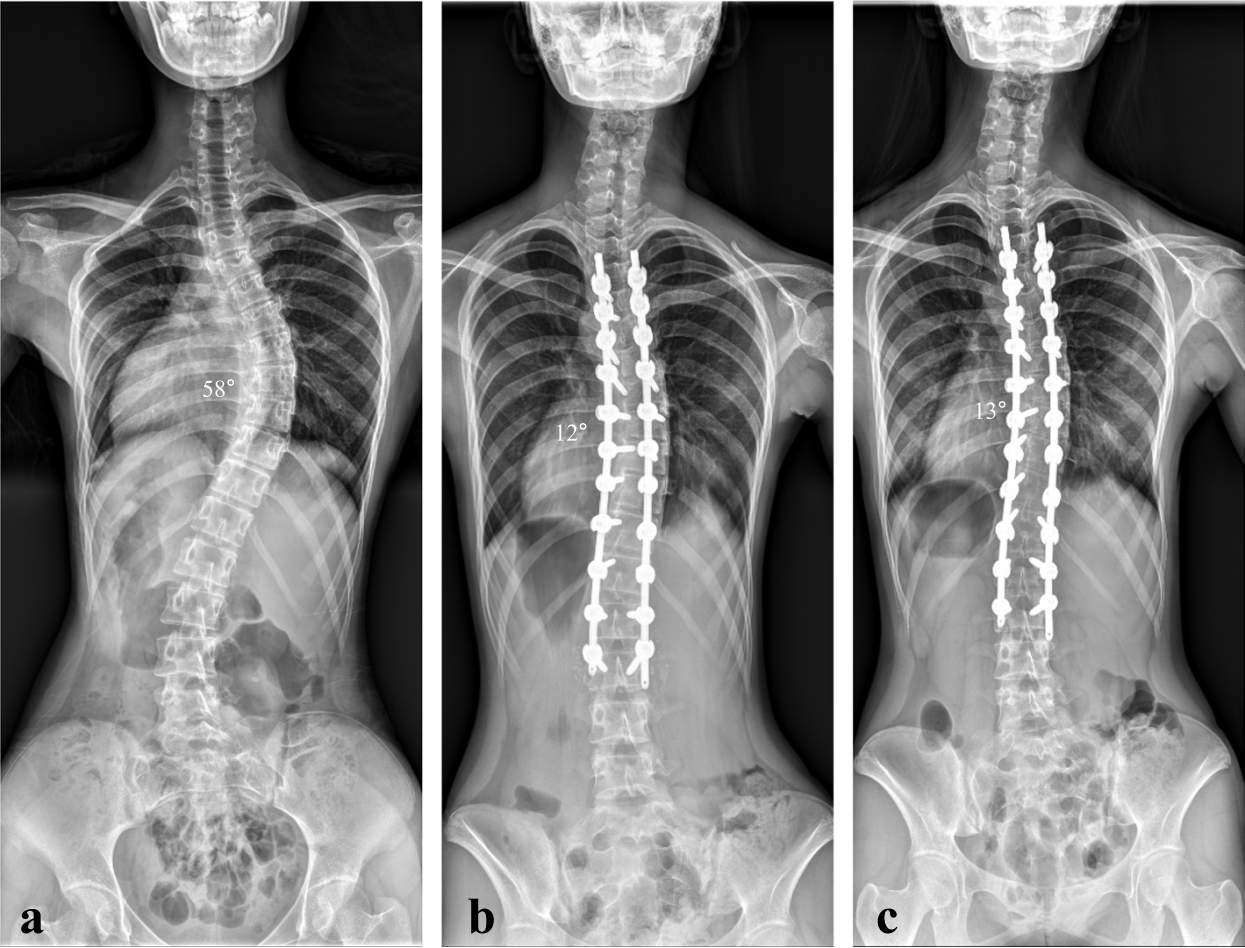
A 15-year-old female patient visited the hospital for progressive spinal deformity. The whole-spine anterior–posterior image showed 58° of scoliosis deformity, and the Risser stage was evaluated as Grade 4 (**a**). Considering the progression of the curvature and the scoliosis deformity of 58°, correction surgery was planned. Using the minimally invasive scoliosis surgery (MISS) technique, correction surgery was performed. Facet fusion with demineralized bone matrix was performed. Postoperatively, the deformity was corrected from 58° to 12° (**b**). On a whole-spine anterior–posterior image taken 2 years after surgery, the reduction was maintained as 13° without reduction loss, segmental fracture, decompensation of the curve, or implant failure (**c**)

The MISS surgical procedure was illustrated as shown. A 3-cm skin incision was used to instrument three to five thoracic vertebrae or three to four lumbar vertebrae. The thoracolumbar fascia was split, and hemostasis was achieved with insulated electrocautery. After sequential dilatation, a 20- to 24-mm diameter tubular retractor system was used to expose the facets and pedicle screw entry points. The facet capsule was destroyed, and the pedicle screw trajectories were traced using the tubular retractor system with a free-hand technique. After inserting the guidewire, the facet joints were reamed with a specially designed facet miller. In the lumbar spine, dissection was performed using the paraspinal Wiltse’s approach. After the guidewire was inserted, the fusion bed was prepared by decortication of the facet joints with a cutting burr. Bone grafts (allograft or graft substitute) were applied over the facet with the help of a 5 cc cut-open syringe and made to settle using an impactor. Pedicle screws were then placed along the guidewires. Contoured rods were inserted in the cephalocaudal direction, and correction was achieved using rod translation and a RD maneuver (Figs. 1 and 2). All patients were prescribed a brace for 3 months.

Preoperative standardized whole-spine erect radiographs (anteroposterior and lateral) were performed in all patients. Supine side-bending views were used to assess curve flexibility, and computed tomography (CT) was used for preoperative planning. Type of curvature, the magnitude of different curves, flexibility, and skeletal maturity were noted. Radiographs were compared to assess curve magnitudes, loss of correction, and implant-related complications. Post-operative CT-scan was performed in patients with back pain and neurological abnormalities only after obtaining consent to tomography. A scheduled CT to evaluate fusion was not performed considering that such imaging is inappropriate due to radiation scattering around the inserted implant and radiation exposure in pediatric patients. Clinical outcomes were evaluated using the Scoliosis Research Society-22 Patient Questionnaire (SRS-22).

Fusion was evaluated using the criteria developed by Bridwell et al. [7]. Fusion was considered “definitive” if trabecular bone was present along the fusion mass on CT or plain radiographs, without evidence of pseudarthrosis (lysis around implants, loss of correction > 10°, or implant breakage). Fusion was considered “probable” if none of the aforementioned features of pseudarthrosis were present, but no fusion mass was visible. “No fusion” was the assigned designation if any of the aforementioned features of pseudarthrosis were present.

The statistical analysis was performed using SAS statistical software (version 8.0; SAS Institute, Inc., Cary, NC, USA). Means and standard deviations were calculated for age, height, weight, body mass index (BMI), follow-up duration, Cobb’s angle, flexibility, SRS-22 scores, and correction rate. A normality distribution test was performed, and unpaired t-tests or ANOVA were used for parametric data. The Kruskal–Wallis test and Mann–Whitney U-test were used for nonparametric data. For categorical variables, King’s classification, Lenke classification, Fisher’s exact test, or the chi-square test with Yates correction was used. Statistical significance was determined at *p* <  0.05.

## Results

A total of 86 patients was included in the study: 43 who underwent COSS and 43 who underwent MISS. In the MISS group, the graft material was allograft for seven patients (Group A), DBM for 21 patients (Group B), and demineralized cancellous bone chips for 15 patients (Group C). All patients were followed for a mean period of 22 months (range, 18–38 months). Patients in the COSS and MISS groups were similar with respect to age, sex, height, weight, BMI, King classification, Lenke classification, and fusion levels. The mean Cobb’s angle improved from 62.3° to 21.3° with a correction rate of 66.1% in the COSS group. In MISS group, the mean Cobb’s angle improved from 63.4° to 25.7° with a correction rate of 59.7%. The difference was not statistically significant (*p* = 0.220). On further follow-up for 24 months, there was a loss of correction of 0.4° in the COSS group and 0.9° in the MISS group (*p* = 0.55) (Table 1).

**Table 1:** Demographic data of the COSS and MISS groups

Variable	COSS Group (*n* = 43)	MISS Group (*n* = 43)	*P*-value
Sex (M:F)	6:37	5:38	0.581^*†*^
Age (years)	14.6 ± 2.4^a^	15.7 ± 2.0^a^	0.352
Height (cm)	157.9 ± 9.0^a^	160.2 ± 5.1^a^	0.328
Weight (kg)	45.1 ± 10.1^a^	48.6 ± 8.1^a^	0.305
BMI	18.2 ± 3.7^a^	18.9 ± 3.1^a^	0.353
King classification (n)			
1	4	5	0.450^*†*^
2	17	9	
3	15	18	
4	3	7	
5	4	4	
Lenke classification (n)			
1	17	18	0.251^*†*^
2	2	3	
3	16	8	
4	1	0	
5	4	10	
6	3	4	
Risser grade	3.4 ± 1.3^a^	3.8 ± 1.4^a^	0.171
Fusion levels	12.3 ± 2.1^a^	11.6 ± 1.2^a^	0.050
Thoracoplasty (No. of ribs resected)	4.6 ± 0.9^a^	4.4 ± 0.9^a^	0.388
Infection	2	2	0.99
Preoperative Cobb’s (^0^)	62.3 ± 15.2	63.4 ± 12.1	0.713
Postoperative Cobb’s (^0^)	21.3 ± 10.4	25.7 ± 8.13	0.029
Final Cobb’s (^0^)	21.8 ± 10.0	26.4 ± 9.5	0.035

*P* < 0.05 is significant

*P*-values are calculated by paired t-test for parametric data and by Mann Whitney U test for non - parametric data

*COSS* Conventional open scoliosis surgery, *MISS* Minimally invasive scoliosis surgery, *n* Number, *M* Male, *F* Female, *BMI* Body mass index

^†^*P*-values are calculated by chi-square test

^a^All values expressed as mean (± standard deviation)

Among the MISS subgroups, patients were similar in terms of sex, age, height, weight, BMI, King classification, Lenke classification, and fusion levels. The mean Cobb’s angle improved in group A from 65.9° to 24.9°, in group B from 61.2° to 24.9°, and in group C from 65.6° to 27.4°. The correction rates obtained were 61.8, 64.3, and 58.4% in group A, B, and C, respectively (*p* = 0.481). There was a loss of 1.8°, 0.1°, and 2.1° in groups A, B and C, respectively, on final follow-up (*p* = 0.153). In group A, a mean of 171.5 mL of allograft was used; in group B, a mean of 57.6 mL of DBM was used; and in group C, a mean of 109.3 mL of demineralized cancellous bone chips was used (*p* <  0.001) (Table 2).

**Table 2:** Demographic comparison between subgroups in the MISS group

Variable	Allograft (Group A) (*n* = 7)	DBM (Group B) (*n* = 21)	Cancellous bone chips (Group C) (*n* = 15)	*P*-value
Sex (M:F)	7:0	19:2	12:3	0.379^†^
Age (years)	15.4 ± 1.8^a^	15.9 ± 2.2^a^	15.6 ± 2.0^a^	0.872
Height (cm)	161.1 ± 5.5^a^	160.7 ± 4.7^a^	159.0 ± 5.5^a^	0.729
Weight (kg)	52.2 ± 8.5^a^	48.9 ± 7.3^a^	45.9 ± 8.6^a^	0.362
BMI	20.2 ± 3.1^a^	19.0 ± 2.8^a^	18.2 ± 3.2^a^	0.450
King classification (n)				
1	1	2	2	0.848^†^
2	1	4	4	
3	4	9	5	
4	0	5	2	
5	1	1	2	
Lenke classification (n)				
1	3	12	3	0.307^†^
2	1	1	1	
3	1	2	5	
4	0	0	0	
5	2	5	3	
6	0	1	3	
Risser Grade	3.6 ± 1.4^a^	4 ± 1.3^a^	3.5 ± 1.5^a^	0.715
Fusion levels (n)	11.8 ± 1.3^a^	11 ± 1.0^a^	12.3 ± 1.1^a^	0.070
Infections	2	0	0	0.020
Preoperative Cobb’s (^0^)	65.9 ± 17.1^a^	61.2 ± 8.7^a^	65.6 ± 11.7^a^	0.462
Postoperative Cobb’s (^0^)	24.9 ± 6.6	24.9 ± 9.4	27.4 ± 7.7	0.633
Final Cobb’s (^0^)	25.2 ± 9.0	24.5 ± 10.4	29.5 ± 8.4	0.291

*P* < 0.05 is significant

*P*-values are calculated by one-way ANOVA test for parametric data and by Kruskal-Wallis test for non-parametric data

*DBM* Demineralized bone matrix, *n* Number, *M* Male, *F* Female, *BMI* Body mass index

^†^*P*-values are calculated by chi-square test

^a^All values are expressed as mean (± standard deviation)

Regarding the fusion rate, final follow-up assessment using CT and X-rays revealed that five and four patients with “definitive” fusion in the COSS group and MISS group, respectively. About 83 and 97% of respective patients in the MISS group and the COSS group showed “definitive” or “probable” fusion, respectively (*p* = 0.07) (Table 3). In the MISS subgroups, group A and group C each had one patient with “definitive” fusion, while two patients in group B had “definitive” fusion. The rates of fusion (definitive + probable) were 85, 100, and 100% in groups A, B and, C, respectively, at the final follow-up (*p* = 0.221) (Table 4).

**Table 3:** Comparison of fusion rates in the COSS and MISS groups

Fusion type	COSS Group (*n* = 43)	MISS Group (*n* = 43)	*P*-value
a	5	4	0.070
b	31	38	
c	7	1	
The rate of fusion (a + b) / (a + b + c)	83.3%	97%	

There was no significant difference in fusion rate (*p* > 0.05)

*P*-values are calculated by chi-square test

a = definitive fusion; b = probable fusion; c = no fusion

*COSS* Conventional open scoliosis surgery, *MISS* Minimally invasive scoliosis surgery, *n* Number

**Table 4:** Comparison of fusion rates in allograft, cancellous bone, and DBM subgroups of MISS

Fusion type	Allograft (Group A) (*n* = 7)	DBM (Group B) (*n* = 21)	Cancellous bone chips (Group C) (*n* = 15)	*P*-value
a	1	1	2	0.221
b	5	14	19	
c	1	0	0	
The rate of fusion (a + b) / (a + b + c)	85%	100%	100%	

There was no significant difference in fusion rate (*p* > 0.05)

*P*-values are calculated by chi-square test

a = definitive fusion; b = probable fusion; c = no fusion

*DBM* Demineralized bone matrix, *n* Number

Regarding mechanical complications, three patients in the COSS group experienced breakage of the distal screw and discontinuity in the distal fusion mass at 10, 11, and 12 months, respectively, after surgery. One patient had loosening of the distal L3 cap after 11 postoperative months in COSS group. Three patients had lysis around the distal screw without any demonstrable fusion mass at the end of follow-up. In the MISS group, only one patient showed a loss of correction greater than 10° in the instrumented segment within eight postoperative months. As for infection, there were two patients in the COSS group who developed infections in the early postoperative period, with one patient developing infection secondary to wound dehiscence. Two patients in the MISS group developed infections, and one of these patients required implant removal because the infection was not controlled by serial repeated debridement. The complication rate was not significantly different between COSS and MISS group (*p* = 0.063).

The clinical outcome assessment at final follow-up using SRS-22 scores revealed that quality of life outcomes were generally better among patients in the MISS group than among those in the COSS group (*p <* 0.001). The scores in the domains of “satisfaction” and “self-image” were significantly better on final follow-up in the MISS group compared with the COSS group (*p <* 0.001). The scores in the domains of “function,” “mental health,” and “pain” did not vary significantly between the two groups (*p* = 0.346, 0.085, and 0.876) (Table 5). Subgroup analysis of the MISS group revealed no significant differences in the SRS-22 scores among groups A, B, and C (*p* = 0.609) (Table 6).

**Table 5:** Clinical outcomes between the COSS and MISS groups

	COSS group (Group A) (*n* = 43)	MISS group (Group B) (*n* = 43)	*P*-value
SRS-22 total	4.4 ± 0.1^a^	4.5 ± 0.1^a^	< 0.001
Functional	4.6 ± 0.2^a^	4.6 ± 0.2^a^	0.346
Pain	4.4 ± 0.3^a^	4.5 ± 0.3^a^	0.085
Satisfaction	4.1 ± 0.1^a^	4.5 ± 0.2^a^	< 0.001
Mental health	4.4 ± 0.3^a^	4.4 ± 0.2^a^	0.876
Self-image	4.3 ± 0.2^a^	4.56 ± 0.3^a^	< 0.001

*P*-values are calculated by paired t-test

*COSS* Conventional open scoliosis surgery, *MISS* Minimally invasive scoliosis surgery, *n* Number

^a^All values expressed as mean (± standard deviation)

**Table 6:** Clinical outcomes of allograft, cancellous bone, and DBM subgroups of MISS

	Allograft (Group A) (*n* = 7)	DBM (Group B) (*n* = 21)	Cancellous bone chips (Group C) (*n* = 15)	*P*-value
SRS-22 total	4.5 ± 0.1^a^	4.5 ± 0.1^a^	4.5 ± 0.2^a^	0.609
Functional	4.8 ± 0.2^a^	4.6 ± 0.2^a^	4.6 ± 0.2^a^	0.560
Pain	4.5 ± 0.4^a^	4.5 ± 0.3^a^	4.6 ± 0.2^a^	0.352
Satisfaction	4.7 ± 0.2^a^	4.5 ± 0.2^a^	4.5 ± 0.3^a^	0.574
Mental Health	4.7 ± 0.2^a^	4.0 ± 0.3^a^	4.5 ± 0.2^a^	0.535
Self-image	4.4 ± 0.2^a^	4.5 ± 0.2^a^	4.5 ± 0.2^a^	0.981

*P*-values are calculated by one-way ANOVA test

*DBM* Demineralized bone matrix, *n* Number

^a^All values expressed as mean (± standard deviation)

## Discussion

Techniques of spinal arthrodesis after deformity correction have been undergoing constant evolution over the last few decades. The fusion rates after COSS varies up to 30% [8, 11, 12]. It was proposed that posterior decortication of the lamina and harvesting of the spinous process enhances fusion rates along with segmental fixation [8]. The facet fusion technique on its own has only been studied by a few authors [17]. Moe et al. observed that radiological pseudarthrosis occurred in 23% of patients after articular facet fusion without instrumentation [18]. With newer instrumentation systems, higher rates of fusion are obtained irrespective of graft material and preservation of midline spinous processes [6, 9]. Recently, Yeh et al. observed that the rates of pseudarthrosis were similar among patients in whom spinous processes were harvested (5.4%) and those in whom the spinous processes were preserved (5.1%) at the end of 24 months [19]. This group observed that patients with preserved spinous processes (*n* = 61) had better pain scores compared with patients (*n* = 43) who had spinous processes harvested. The group concluded that using spinous processes as a source of local autologous bone graft material is not necessary. The pain scores were better when spinous processes were preserved. Twenty-six percent (16/61) of patients in the spinous process harvesting group required pain medications, while only 9% (4/43) of patients with preserved spinous processes required pain medications. In our study, even though there was no statistically significant difference between MISS and COSS groups, the fusion rate was better in the MISS group. In addition, through the SRS-22 questionnaire, we confirmed that the results of MISS surgery were higher in satisfaction than those of COSS surgery. In view of the results of our study and the surgical results of Yeh et al., facet fusion using pedicle screws in scoliosis surgery could induce a “potential” fusion grade that, is sufficient for fusion in immature AIS patients.

MIS techniques have been successfully adopted for adult deformity correction and, sporadically, for pediatric deformity correction [20]. The perioperative advantages of minimal blood loss, smaller incision, lower infection rate, and faster recovery have encouraged increasing number of surgeons to adopt MISS techniques [16, 17, 20, 21]. In our previous study, MISS techniques have a merit for hospital stays compared to COSS (12.0 days in MISS vs 16.2 days in COSS, *p* <  0.001) but MISS requires longer operative times (441 min in MISS vs 287 min in COSS, *p* <  0.001) [21]. However, the fusion rates using different graft materials in spine surgery have not been widely studied. The fate and strength of fusion are important to know as the bed for fusion is provided by the facets and not by spinous process and lamina.

In a previous study, facet fusion was studied in the context of MISS, and no case of pseudarthrosis was reported. However, the number of cases was small (*n* = 7) and rhBMP-2 was used for fusion [16]. We believe that rhBMP-2 has frequent side effects; moreover, there are no data supporting its long-term safety. Hence, rhBMP-2 should not be used in adolescents. The selection of appropriate graft materials for MISS remains a major concern. We wanted to avoid iliac crest grafting for several reasons. Donor site pain, bleeding, neurologic injury, hernia, fracture, and blood loss are common after autologous iliac crest harvesting, with reported rates ranging between 19 and 31% [22]. Skaggs et al. observed that posterior iliac crest bone grafting in spine surgery in 87 children was associated with significant donor site pain in 24% of patients and limitation of daily activities in 15% of patients even 4 years after surgery. Freeze-dried corticocancellous allografts were used for COSS and some cases of MISS [23]. Higher rates of fusion equal to those of autologous grafts have been reported by several authors and has shown no donor site complications [24, 25]. In our study, we observed pseudarthrosis rates of 16.3 and 3% after COSS and MISS, respectively. Despite no statistical differences of pseudarthrosis rate, COSS performs posterior fusion that requires large amount of graft volume and large area decortication compared to facet fusion in MISS. The facet fusion only needs to small amount of graft volume in the facet area, which contribute to sufficient fusion rate.

Knapp et al., in their study of 111 AIS patients using various types of instrumentation techniques along with allografts, observed a mean loss of correction of 5.9% and a pseudarthrosis rate of 2.7% [9]. Theologis et al., in their study of over 461 patients, found no cases of pseudarthrosis out of 199 patients with allograft after 2 years of follow-up [12]. Price et al. found a significantly higher failure rate of 28% with allografts compared to 13% with the use of iliac crest bone grafts and 11.1% with composite grafts in AIS surgery [11]. One of notable results by Price et al. was that pseudarthrosis was not significantly affect pain and implant-related complications. In our study, pain in clinical outcomes, correction loss, and complications was not statistical differences between facet fusion in MISS and posterior fusion in COSS. Therefore, facet fusion in MISS also provide comparable postoperative outcomes as much as posterior fusion in COSS.

Another concern is the high infection rates associated with allografts. All four infections observed in our study were associated with the use of allografts. Reported incidences of bacterial infection after all forms of allograft range from 4 to 13% [25]. However, the risk depends on the patient’s primary condition, operation time, and associated skin complications [26]. There is a potential risk of disease transmission with allografts, but this risk is exceedingly low. As reported by Asselmeier et al., no such cases of disease transmission have been observed since 1951 [27]. The risk of transmission with freeze-dried allografts is much less compared with that associated with fresh-frozen allografts [27]. Hence, we prefer to use freeze-dried corticocancellous allografts. From our study, the preliminary outcomes suggest that selection of facet fusion and of graft material produce similar results to COSS. However, the rate of infection was higher with the use of allografts.

Several graft substitutes have been studied, and many have shown encouraging results [9]. We wanted to analyze a graft material that possessed both osteoinductive and osteoconductive properties in order to enhance the rates of fusion using as little graft material as possible. Demineralized matrix has mainly osteoinductive properties, with some products having osteoconductive properties, as determined by the methods of preparation and sterilization [28]. DBM is an organic derivative of allograft used in surgery for AIS [6, 12]. DBM can be processed as granules, powder, or chips from human cortical or corticocancellous bone. Terminal sterilization with irradiation, ethylene oxide, glutaraldehyde, and formaldehyde has been shown to reduce the osteoinductive properties of DBM [6]. We used two types of demineralized bone: a matrix form and a cancellous chip form. The selection of two different forms was inspired by variation in rates of fusion observed in studies due to the manner in which DBM was prepared and the carrier with which DBM was combined [3].

All mechanical failures of COSS were associated with a failure of formation of a solid fusion mass at the distal end of the construct. Several authors have reported the distal extent of fusion to be troublesome. Knapp et al. observed three cases of pseudarthrosis in his series of 111 patients and noted that two of three patients had dislodgement of the distal two hooks at intervals of 4 months and 1 year after surgery, respectively [9]. Yeh et al. had one patient who developed bilateral L4 screw loosening at the end of 24 months and another patient with L3 screw breakage at the end of 19 months [19]. In the group with preserving spinous process, one patient had right L1 screw breakage at the end of 50 months, and another had left L4 screw cap loosening at the end of 27 months [19]. Betz et al. observed one case of pseudarthrosis, 12 months after surgery, with distal screw breakage at L4 and caudal fusion mass formation [6]. However, most rod breakage occurs in the first 2 to 3 years after implantation [9]. Investigations of the long-term outcomes of the newer techniques are needed for further comparison.

We also analyzed the amount of graft material used in different groups. Since different graft materials were used, a statistical comparison was inappropriate, but the amount of graft substance used for MISS was much less than that used for COSS.

Our study had a few limitations. Radiographs were used to evaluate fusion for most of the patients. Radiographs, including bending films, are helpful to rule out instability but have their own limitations. An interobserver agreement rate of 100% with the fusion criteria has been reported [10]. Even though the main limitation is variability between observers, fusion tends to be over-reported (false negative for non-unions) overall. CT is currently the investigation method of choice to confirm or rule out pseudarthrosis. Radiation exposure after a single thoracolumbar CT scan is 10 to 40 times greater than that of a standing whole-spine x-ray, and this increases cancer risk by 0.32 to 0.52% per CT scan. Subjecting asymptomatic patients to such amounts of radiation would be unethical. CT scan should be considered in symptomatic patients (those with axial back pain or radiological signs of pseudarthrosis), however, for confirmation and preoperative planning of revision surgeries. Furthermore, we instrumented all levels in the fusion mass. Hence, we had higher implant density indices (> 2) with better curve correction, and the loss of correction over time should have been minimal. However, since MISS is a novel technique associated with patient satisfaction, and the fusion rates with this technique have not yet been determined, we will continue to instrument all levels until long-term outcomes have been established. Another limitation was the relatively small number of patients; we did not perform a power analysis to determine the number of cases to be included in each group and subgroup before the study. Finally, our follow-up should be considered a preliminary analysis as pseudoarthrosis has been reported to develop as late as 6 years after scoliosis surgery.

## Conclusion

MISS showed comparable fusion rates and clinical outcomes to those of COSS. Furthermore, the type of graft substitute among allograft, demineralized bone matrix, and demineralized cancellous bone chips did not affect facet fusion rate or clinical outcomes in the treatment of AIS using MISS.

## Data Availability

The datasets used and/or analyzed during the current study are available from the corresponding author on reasonable request.

## Abbreviations

MISS: Minimally invasive scoliosis surgery
AIS: Adolescent idiopathic scoliosis
COSS: Conventional open scoliosis surgery
DBM: Demineralized bone matrix
MIS: Minimally invasive surgery
RD: Rod derotation
CT: Computed tomography
SRS-22: Scoliosis Research Society-22
BMI: Body mass index
